# Progress of Edge-Emitting Diode Lasers Based on Coupled-Waveguide Concept

**DOI:** 10.3390/mi14061271

**Published:** 2023-06-20

**Authors:** Lili Han, Zhaowei Wang, Nikita Yu. Gordeev, Mikhail V. Maximov, Xiansheng Tang, Artem A. Beckman, Grigoriy O. Kornyshov, Alexey S. Payusov, Yuri M. Shernyakov, Alexey E. Zhukov, Kuilong Li, Ruizhan Zhai, Zhongqing Jia, He Yang, Wei Zhang

**Affiliations:** 1 Laser Institute, Qilu University of Technology (Shandong Academy of Sciences), Jinan 250104, China; 2 Ioffe Institute, St. Petersburg 194021, Russia; 3 Nanophotonics Laboratory, Alferov University, St. Petersburg 194021, Russia; 4 International Laboratory of Quantum Optoelectronics, National Research University Higher School of Economics, St. Petersburg 190121, Russia; 5 School of Instrumentation and Optoelectronic Engineering, Beihang University, Beijing 100191, China

**Keywords:** diode lasers, CLOC, waveguide, high power

## Abstract

Semiconductor lasers have developed rapidly with the steady growth of the global laser market. The use of semiconductor laser diodes is currently considered to be the most advanced option for achieving the optimal combination of efficiency, energy consumption, and cost parameters of high-power solid-state and fiber lasers. In this work, an approach for optical mode engineering in planar waveguides is investigated. The approach referred to as Coupled Large Optical Cavity (CLOC) is based on the resonant optical coupling between waveguides and allows the selection of high-order modes. The state-of-art of the CLOC operation is reviewed and discussed. We apply the CLOC concept in our waveguide design strategy. The results in both numerical simulation and experiment show that the CLOC approach can be considered a simple and cost-efficient solution for improving diode laser performance.

## 1. Introduction

The global laser market steadily grows. A significant part of the market is semiconductor lasers, most of which are represented by high-power edge-emitting lasers due to their compactness, reliability, high efficiency, and high level of technology development. Currently, the development of solid-state laser technology is largely determined by progress in improving the performance of pumping laser diodes. Even in high-power industrial laser systems, more reliable and compact diode-pumped lasers are intensively replacing lamp-pumped lasers. High-power diode lasers that appeared in the late 80s allowed the creation of compact diode-pumped units with output power as high as 10 kW. The high quantum efficiency and narrow spectral line allow selective pumping into the absorption line of the active element with optical efficiency exceeding 50%. Thus, the use of semiconductor laser diodes is presently considered the most advanced option for achieving an optimal combination of efficiency, energy consumption, and cost parameters for high-power solid-state and fiber lasers. 

Cost is one of the main factors affecting the use of high-power diode lasers. One watt of the laser output optical power (dollars-per-watt, $/W) in the typical 9xx nm waveband cost more than US$100 20 years ago and now approaches US$1 [1]. A prime cost is fixed by the manufacturing technologies of both the laser chips themselves and the laser modules. The maximum continuous wave (CW) optical power delivered by commercially available single lasers lies in the range of 10–20 W for a 100 µm light emission aperture, depending on the emitting wavelength. One method to increase the power of diode laser modules is to combine several lower-power oscillators to create a higher-power beam. While that strategy does increase the overall power, it does not reduce $/W. A natural way to reduce $/W and increase the market attractiveness of high-power diode lasers is to enhance the maximal output optical power of a laser diode while keeping the laser cost unchanged. In principle, it can be performed by waveguide engineering, which is an effective approach for simultaneously improving optical, electrical, and thermal characteristics. Particularly it makes it possible to increase the output power by enlarging the laser aperture. 

In this paper, we review our approach for designing transverse waveguides of broad-area lasers, which are referred to as coupled large optical cavities (CLOC). We briefly discuss existing trends in developing laser waveguides, present our general waveguide design strategy, describe the physical principles of the CLOC operation, show the implementation of the concept, and finally discuss some aspects related to the CLOC application for other types of diode lasers.

## 2. Trends in Designing Edge-Emitting Laser Waveguides

There are thermal and non-thermal limitations on the optical power of diode lasers. The diode laser parameters affect the output performance in a rather complicated manner. Fundamentally, the output optical power of an edge-emitting laser depends on the electrical-to-optical power conversion efficiency (PCE), which determines how much of the electrical power is wasted on heat. The main Joule heat comes from the active region and is dissipated into a heatsink. Semiconductor layers located between the active region and the laser surface have higher thermal resistance, which prevents effective heat extraction and can cause significant overheating. The self-heating decreases the laser PCE and the output optical power, therefore. Other heat-induced effects are wavelength redshift, thermal lensing, and filamentation. The latter two processes significantly worsen the laser beam quality and can trigger catastrophic optical mirror damage (COMD). The temperature-induced red-shift in GaAs-based lasers has a rate of 0.3~04 nm/K (it depends in particular on the central wavelength). When lasers are used as pumping sources, a temperature-induced wavelength mismatch between laser and pumped media can dramatically decrease the pumping efficiency. High series resistance contributes to self-heating and, at the same time, decreases the laser PCE due to the parasitic voltage drop. The largest portion of up to 40% of the total diode laser electric resistance is considered to come from p-claddings [2]. The nonradiative recombination also contributes to the unwanted power loss mainly associated with heating. The internal efficiency reflects how effectively the injected current is converted into photons. Internal efficiency depends on the laser parameters in a complicated manner. The most influential ones are the crystal quality of the laser wafer, active region band structure, and optical waveguide design. Improperly designed active regions and waveguides can cause parasitic recombination [3]. The internal optical loss is associated mainly with the free-carrier absorption and increases, therefore, when the optical mode significantly overlaps with highly doped layers, especially p-doped one’s [2]. A current-induced accumulation of carriers in the initially undoped waveguide layers can also significantly increase internal optical loss. High internal optical loss increases the threshold current, contributes to the self-heating and therefore significantly reduces the total output optical power.

In developing high-power diode lasers, one should remember that besides the output optical power, the other critical parameters are the beam divergence and quality, spectral width, reliability, and manufacturing cost. 

In summary, the strategy of increasing the maximal output optical power of diode lasers should be focused on the following.

- Improvement of the heat dissipation from the active region of diode lasers due to the use of heatsinks with high thermal conductivity and reducing the thermal resistance by means of reducing the distance from the active region to the heatsink surface.
- Reducing the electric series resistance by using thinner p-claddings and undoped layers.
- Reducing the laser internal optical loss mainly by decreasing overlap of the optical mode with highly doped layers.
- Protection of laser mirrors from COMD by applying dielectric coatings, passivation and reducing the optical power density.
- Increase the focusing efficiency of the laser beam by reducing its divergence.
- Effective suppressing of the high-order lateral modes that may arise due to diode overheating and deteriorated lateral beam quality.

In 2004, a team from Ioffe Institute (St. Petersburg, Russia) [4] used a broadened optical waveguide in 1.06 µm high-power lasers, which allowed effective confinement of the laser mode within the undoped layers and, thus, reduced the optical loss down to 0.34 cm^−1^. A quantum well active region was shifted from the waveguide center. Due to this shift, the second-order vertical optical mode has a reduced optical confinement factor and does not contribute to the lasing. CW optical power is as large as 16 W was obtained from 100 µm-wide stripes. The broadened waveguide reduced the intensity at the facet and avoided the COMD damage. The disadvantage of this approach is that the design is limited in terms of the further waveguide broadening and is too sensitive to current and temperature changes. However, the general idea of broadening the optical waveguide has evolved into the Super Large Optical Cavity (SLOC) [5] design intensively developed by Ferdinand-Braun-Institute FBH (Berlin, Germany). The approach allows increasing the laser waveguide thickness up to 10 µm providing the vertical beam divergence as narrow as 15 deg. This waveguide was originally a multimode waveguide. To suppress high-order modes, the laser claddings have reduced thickness as thin as 0.4 µm. As a result, high-order modes couple to the highly-doped contact layer and the substrate and experience extra optical losses. The fundamental mode has a low optical loss and allows making the cavity length as long as 8 mm to provide high optical power. The fundamental mode profile has a Gaussian shape which positively affects the laser beam quality. The approach still has some disadvantages. Due to the thermal and current-induced changes of the waveguide and claddings refractive indices, in some cases, high-order modes may contribute to the lasing. In 2012 the FBH team showed the maximal CW optical power of 25 W presented in [5]. The lasers have shown moderate operation time of fewer than 5000 h. In [5], they have also discussed limitations to peak power. At high current densities, band-bending in the p-side waveguide leads to a very large concentration of carriers accumulated in this region. This effect results in power saturation caused by the current-induced optical loss, which can be considered a major drawback of SLOC lasers. The fundamental issues of this phenomenon were theoretically studied in [3]. The authors proposed a strongly asymmetric laser waveguide allowing shifting of the active region toward the p-cladding and, thus, dramatically reducing the optical loss [6]. Since that time, a trend for using asymmetric, not very broad waveguides has been intensively developed. 

The FBH team has also developed a similar concept which has evolved into the structures termed by the authors as Extreme Double Asymmetric (EDAS) [7]. The name reflects that the structure design possesses a highly asymmetric cladding layer composition and waveguide thickness. The thin p-side waveguide provides low series resistance, low internal optical losses, and low bias-driven leakage currents. Low optical losses are attributed to the small overlap of the fundamental vertical mode with the p-waveguide [8]. The team has reported CW optical power exceeding 15 W. Further improvement of the concept has resulted in the extreme-triple-asymmetric (ETAS) laser design [9,10]. A third asymmetry in the graded profile of the refractive index for the layers on either side of the quantum well allows fine-tuning of the optical field. The ETAS lasers having 100-μm wide aperture emitting at 940 nm have shown up to 63% power conversion efficiency at 14 W CW optical output power [9]. It is important to note that maximal output optical power can be increased by using a larger laser-emitting aperture [1]. A team from Fujikura Ltd. (Chiba, Japan) has reported 30 W optical power in lasers with ~200 µm aperture [2]. Such a large aperture is possible due to the effective heat dissipation resulting from using an Asymmetric Decoupled Confinement Heterostructure (ADCH), another variation of asymmetric waveguides. 

The world leaders in manufacturing high-power diode lasers are Lumentum (Milpitas, CA, USA), known as JDSU before 2015, NLIGHT (Vancouver, WA, USA), and IPG (Oxford, MI, USA). Driven by the increasing power of fiber lasers, diode lasers with both high power and high brightness are being intensely developed to achieve high performance and reduced manufacturing costs. Lumentum has reported 27 W CW optical power from 100 μm aperture 4 mm long lasers emitting in 910–980 nm wavelength range [11]. The company associates high output power with improved PCE. Better heat dissipation reduces thermal lensing and decreases the blooming of the far-field divergence at high pumping currents. In 2017, IPG showed CW optical power as high as 30 W [12] from 976 nm lasers having a 5-mm cavity length. The company claims that reduced ohmic and thermal resistances, combined with improved linearity of light-current characteristics, have improved the PCE with the current. NLIGHT has introduced a new design of broad-area lasers and shown 25 W diodes for coupling into 105 μm fiber [13]. These companies have revealed no detailed information on the laser designs they used. However, in their publications, they reported to have used modified laser wafer designs and referred to the papers describing asymmetric-waveguide approaches, which is indirect evidence of using this concept. 

The main disadvantages of the strongly asymmetric waveguide relate to a very low refractive index cladding-waveguide contrast at the n-side. It is made intentionally to provide high-order mode selection, namely for promoting the fundamental mode lasing. However, due to this low contrast, the fundamental mode has a large exponential decay on the n-side and its profile can be extremely sensitive to the pumping currents and temperature rise caused by the laser self-heating. Transverse mode profile instability can result in blooming the vertical divergence and in worsening the beam quality. One more drawback is that the epitaxial growth of low Al material is rather challenging because of the difficulty of precisely controlling so small Al mole fractions (a few percent). Additionally, this approach is hardly applicable to Al-free materials. On the opposite p-side, the waveguide contrast is rather high, which should prevent the lasing mode from penetrating into the highly doped p-cladding. High contrast is usually provided by higher Al fraction in AlGaAs claddings, which in turn worsens the structure’s thermal resistance. The high Al fraction may cause difficulties in effective p-doping. No information is available about the application of this method to 808 nm laser structures. In summary, despite the fact that asymmetric waveguides make it possible to obtain high optical power, the approach has significant drawbacks and can hardly be adapted for lasers of a different spectral range.

## 3. Single and Multimode Waveguides

**Figure 1:**
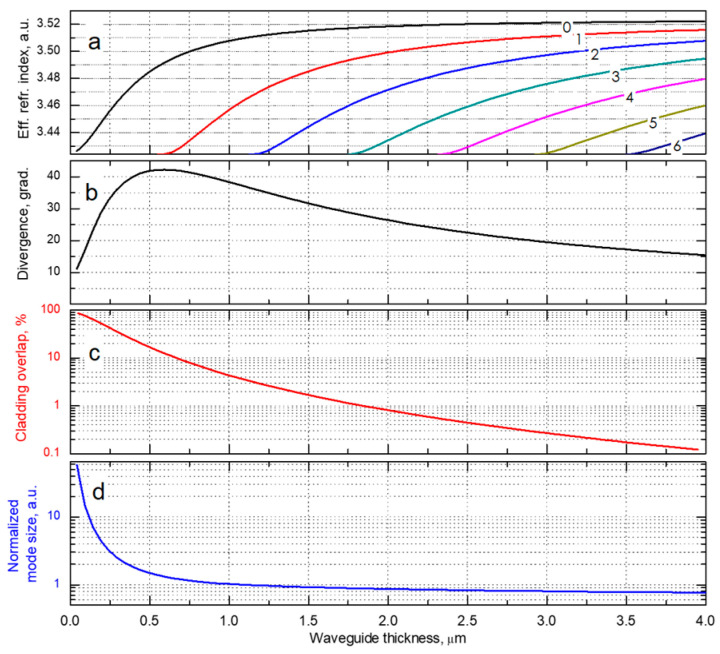
Dependence of the effective refractive indices (**a**), the fundamental mode beam divergence FWHM (**b**), mode-claddings overlap (**c**), and normalized mode size at the 1/e2 level (**d**) on the waveguide thickness. The modeled waveguide is symmetric with GaAs core and Al_0.15_Ga_0.85_As claddings. The wavelength is 980 nm.

Generally, a laser waveguide consists of a high refractive index core surrounded by two low refractive index claddings. Thicknesses and refractive indices of the core and claddings define the guided optical modes and their effective refractive indices (Figure 1a) [14] p. 50: N≡β/k, where *β* is the propagation constants, and k is the wavenumber in a vacuum. Figure 1b–d shows a typical evolution of the fundamental mode parameters against the waveguide thickness. 

When the waveguide is relatively narrow, the fundamental mode has large exponential decay in the claddings resulting in the non-Gaussian intensity profile accompanied by large mode overlap with lossy, highly doped claddings [15]. With increasing the thickness, the fundamental mode becomes more localized within the waveguide core, so the mode-cladding overlap substantially reduces (Figure 1c). Starting from a certain waveguide thickness, the mode size is almost equal to it, follows this value (Figure 1d), and does not depend on the waveguide index steps. Thereby temperature-induced and current-induced refractive index variations would only slightly affect the fundamental mode size and, correspondingly, its far-field pattern behavior. In the simulated waveguide, its thickness range favorable for the fundamental mode stability lies above approximately 1.5 μm. Having looked at the dispersion curves in Figure 1a, one can see that the waveguide of this thickness guides several optical modes. This consideration is relevant to asymmetric multimode waveguides as well. Our waveguide design strategy is built upon ensuring fundamental mode lasing by eliminating high-order modes in naturally multimode waveguides [15]. We should note that “multimode waveguide” does not necessarily mean a broadened one. It can be rather narrow.

## 4. CLOC Operation

**Figure 2:**
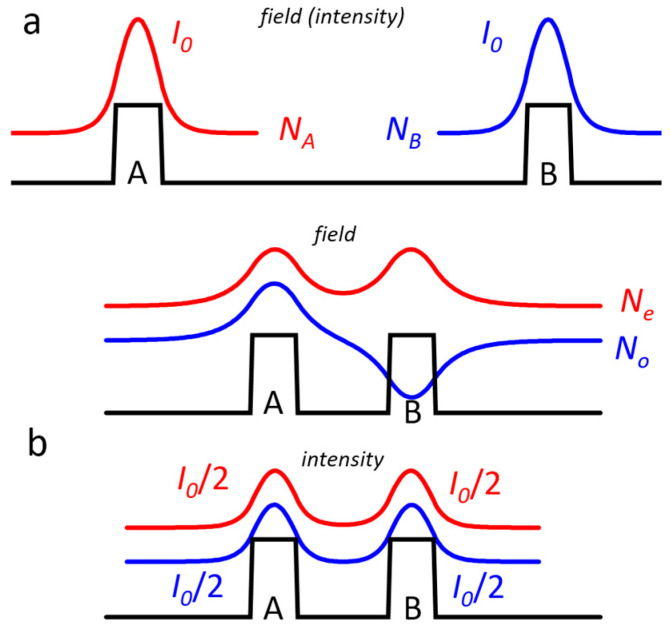
Mode profiles in identical codirectional waveguides: (**a**) two solitary waveguides; (**b**) two coupled waveguides.

Our waveguide design strategy has been realized by using the CLOC concept. It is called after the physical effect that it exploits, namely, optical coupling. Let us consider two identical codirectional optical single-mode waveguides, A and B (Figure 2). If the distance between the waveguides is rather large, then each operates independently. Their guided modes are identical (Figure 2a) and characterized by effective refractive indices N_A_ and N_B_. The optical coupling takes place if the distance between the waveguides decreases (Figure 2b) because the electromagnetic field of one waveguide is perturbed by another waveguide. As a result, in this waveguide system, new optical modes, often referred to as composite, are formed. In the considered identical A and B waveguides, these odd and even modes have different field profiles and, consequently, different effective refractive indices but almost identical intensity distributions. It is important to note that each of the modes is equally distributed between the waveguides. It means that if, for instance, a solitary waveguide A has a guided mode with intensity I_0,_ then in the coupled waveguide A, this mode transforms and has two times lower intensity I_0_/2. Coupling between the waveguides is substantial if the optical modes in the solitary waveguides have equal or nearly equal effective refractive indices N_A_ = N_B_ [16].

**Figure 3:**
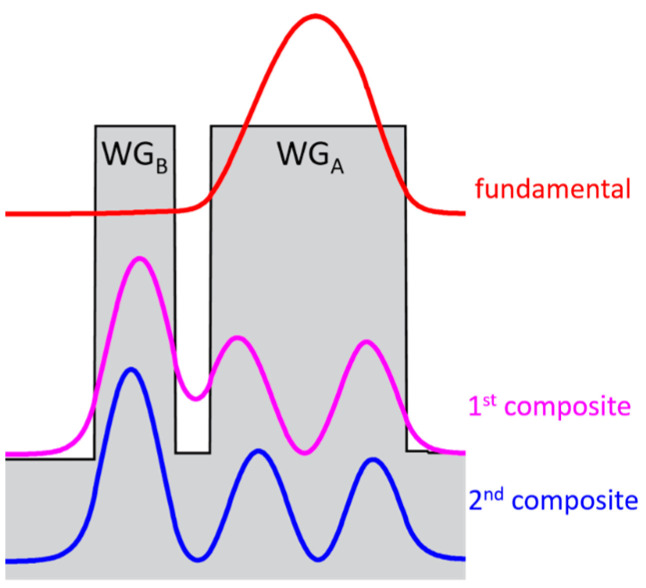
Intensity profiles of the fundamental and two composite modes in two coupled waveguides having different thicknesses.

Since these indices are the mode parameters, we can construct a system containing a multi-mode waveguide WG_A_ optically coupled with a single-mode one WG_B_ in such a way that the index equality is fulfilled for a high-order eigenmode of the WG_A_ and the only eigenmode of the WG_B_ (Figure 3). As a result, two composite modes are formed, and each of them has half the intensity of the high-order eigenmode. This idea is employed in our CLOC laser concept so that the multi-mode waveguide contains an active region, and a coupled single-mode waveguide is passive and doped to pass electric current. A selected high-order mode of the active waveguide tunnels into the passive one forms two composite modes. The CLOC lasers make use of two mode-suppression mechanisms. The first one is the reduced mode intensity and gamma factor in the active waveguide; the second one is the high optical loss originating from the mode overlap with the doped passive waveguide core.

**Figure 4:**
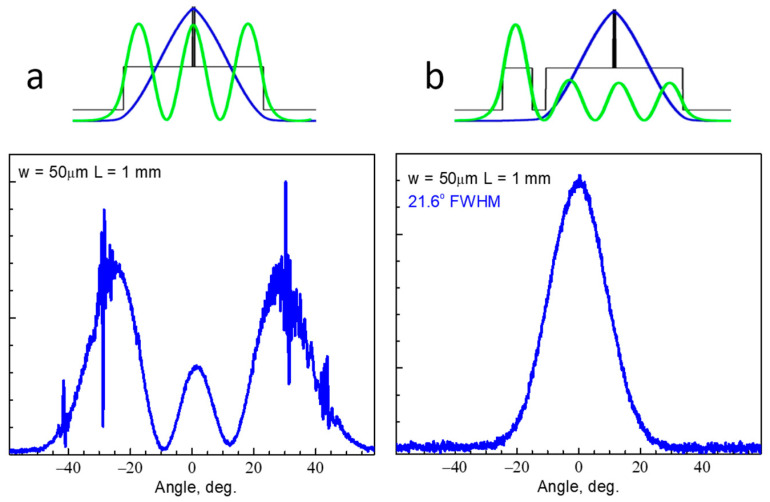
Mode intensity profiles and transverse far-field patterns of the high-order mode and fundamental mode for the reference (**a**) and CLOC (**b**) lasers, respectively.

Initially, the CLOC concept has been proposed for broadening transverse laser waveguide via suppressing parasitic high-order mode. In our proof-of-concept experiment [17], we have compared CLOC lasers and reference ones. Both InGaAs (λ ≈ 1.04 µm) quantum well lasers had identical 2.5 µm-thick undoped GaAs waveguides (Figure 4). The only difference is that the CLOC structure had an extra n-GaAs passive waveguide separated from the active waveguide with an n-AlGaAs layer. Fundamental modes in both structures have identical profiles. The major difference between the two lasers is the absence of a second-order mode in the CLOC structure. Both CLOC and reference lasers showed similar parameters (threshold current, efficiency, spectra). However, over the entire pumping current range, the CLOC lasers have shown stable single-mode emission (Figure 4b), while the reference lasers emitted on high-order modes (Figure 4a).

**Figure 5:**
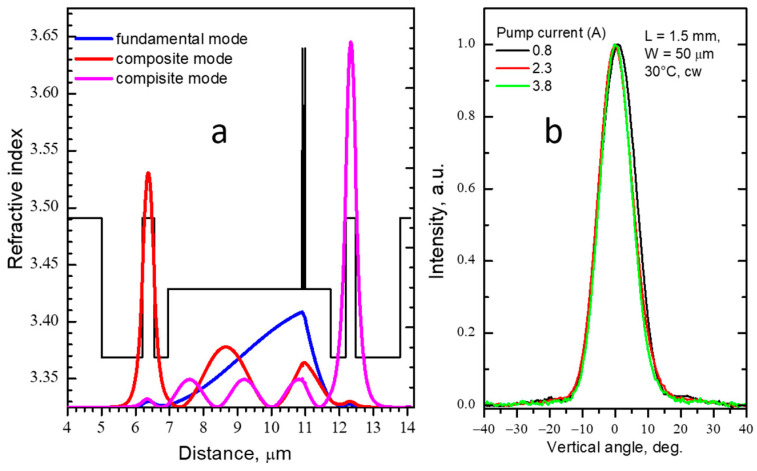
The “1 + 2” CLOC laser: (**a**)—refractive index profile and simulated intensity distributions of the fundamental mode; (**b**)—far-field patterns measured in CW regime at different currents.

The CLOC structures can have a more advanced design, “1 + 2”, where two different passive single-mode waveguides are placed by either side of the active waveguide (Figure 5a). In this case, each parasitic high-order mode tunnels into the corresponding passive waveguide. We used this approach in designing a waveguide as broad as 4.8 μm [18]. The partially doped Al_0.1_Ga_0.9_As active waveguide containing an InGaAs quantum well active region was optically coupled with two GaAs passive waveguides with the thicknesses of 310 nm and 375 nm, which allowed for suppressing the first and second transverse modes. Measurements of the transverse far-field patterns in the CW regime have shown that the single mode remains stable throughout the entire pumping current range (Figure 5b). The obtained divergence is 13 ± 1 deg. FWHM was very close to the diffraction limit for the 4.8 μm emitting aperture. The stability of far-field patterns was measured in the temperature range from 20 to 90 °C in the pulsed mode. No pronounced changes in the beam divergence were observed. These measurements confirm that in multimode-broadened CLOC waveguides, temperature- and current-induced refractive index variations weakly alter the fundamental mode size and, correspondingly, its divergence [19].

For high-power lasers [20,21,22], broadened waveguides have an advantage since they reduce optical power density on the laser facets and, thus, may prevent COMD. However, the COMD threshold also can be successfully increased by using coating and passivation for the facets [23]. The narrower vertical divergence associated with the broadened waveguide is not crucial for edge-emitting lasers. We should note that using broadened waveguides in high-power lasers has some weaknesses. A larger p-side waveguide core increases optical loss and series electrical resistance. These problems can be tackled by shifting the active region toward the p-cladding. However, this approach results in the natural reduction of the optical confinement factor and sometimes promotes high-order mode lasing. The former would require using a multilayer active region. 

In connection with the above, the CLOC concept can be further updated for high-power lasers. The basic idea is that the laser waveguide is multimode and rather narrow (1–1.5 μm), and the active region is extremely shifted toward the p-cladding. Parasitic high-order modes are eliminated by means of the CLOC. This approach has some benefits:

- The multimode waveguide ensures strong fundamental mode localization within the core, which reduces the optical losses in the claddings;
- For the same reason, a thinner p-cladding can be used, which decreases thermal and electrical resistances;
- Both rather narrow waveguides and shifted active regions reduce optical loss in the p-side of the waveguide core and result in lower thermal and electrical resistances.

**Figure 6:**
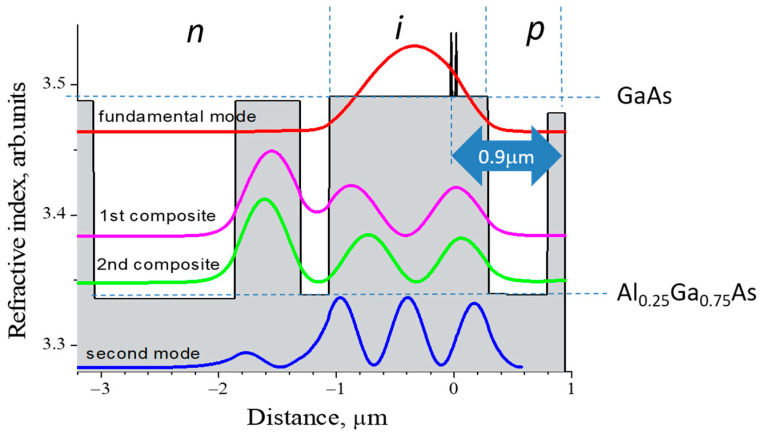
Refractive index and optical mode profiles of the CLOC laser with shifted active region and thin p-cladding.

We have experimentally realized this concept in CLOC lasers possessing the GaAs waveguide with a thickness of 1.35 µm [24]. The wafer was grown by MOCVD on an n + -GaAs substrate. Besides the active waveguide, it contains (Figure 6) a passive one with a thickness of 0.55 µm introduced to suppress first-order mode. The active region based on two InGaAs QWs (wavelength ~1 µm) is shifted by 380 nm from the active waveguide center toward the p-Al_0.25_Ga_0.75_As cladding. The latter has a reduced thickness of 0.5 µm. In this regard, it should be noted that a thinner p-cladding layer significantly improves the electrical resistance of the laser heterostructure. For example, considering an AlGaAs cladding layer with a hole concentration of about 5 × 10^17^ cm^−3^ and corresponding hole mobility of about 90 cm^2^/(V × s) [25], a decrease of the layer thickness from 2 to 0.5 µm results in a decrease of the resistance from 2.8 × 10^−5^ Ω × cm^2^ (while the total electrical resistance of the whole structure is typically 10^−4^ Ω × cm or slightly less) to 0.7 × 10^−5^ Ω × cm^2^.

The active region is located near the minimum of the second-order mode, which prevents its lasing. The laser wafer is completed by a p-GaAs contact layer (0.15 µm), so the active region is situated at a depth of only 0.92 µm from the wafer surface. The design allowed us to achieve an internal optical loss as low as 0.4 cm^−1,^ and shallow active region location has resulted in low thermal resistance of 6.0 (K/W) × mm. The lasers have shown a divergence of 34 deg. Almost unchangeable in the temperature range of 20–80 °C and the cw output power exceeding 12 W [19].

**Figure 7:**
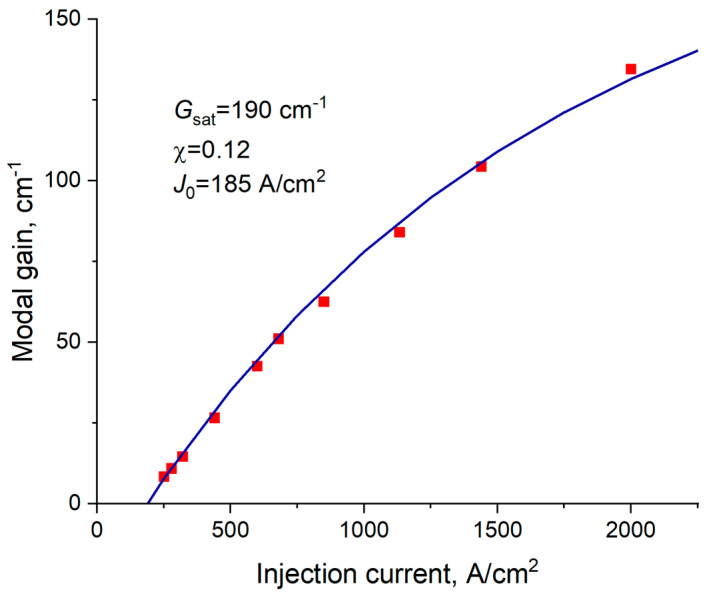
Modal gain against injection current for 5-times stacked InGaAs quantum well-dots: symbols-experiment, line-fit with an exponential-based function.

It is worth emphasizing that the feasibility of the CLOC concept is significantly supported by the high optical gain achievable by the active laser region. This is because shifting the mode position towards the p-type doped cladding layer, in combination with the extended mode profile, strongly decreases the optical confinement factor so that higher material gain is required to balance the total optical loss. An excellent candidate for exploitation in the CLOC lasers is so-called quantum well-dots, which represent very dense arrays of InGaAs quantum islands formed by deposition on slightly misoriented GaAs substrates [26]. The maximal material gain was shown to exceed 10^4^ cm^−1^. As an example, Figure 7 demonstrates the modal gain *G* as a function of the injection current density *J* for the fivefold stacked array of InGaAs quantum well-dots. The gain data were evaluated as the sum of the output loss, and the internal loss measured for the stripe lasers with cavity lengths varied from 100 to 2000 µm. The experimental data can be fitted well with the following equation: $G=G_{sat}\left[1-exp(-\chi (J-J_{0})/J_{0})\right]$, where $G_{sat}$ stands for the saturated gain, $J_{0}$-transparency current density, χ-non-linearity parameter. The saturated gain, i.e., the maximal achievable, is about 190 cm^−1^, which corresponds to approximately 40 cm^−1^ per single InGaAs plane in the active laser region.

## 5. Expansion of CLOC Approach

In this section, we discuss some aspects concerning expanding the CLOC concept beyond transverse waveguides in broad-area edge-emitting lasers, namely:

- narrow-ridge lasers;
- edge-emitting lasers based on lateral CLOC waveguide;
- microdisk lasers.

**Figure 8:**
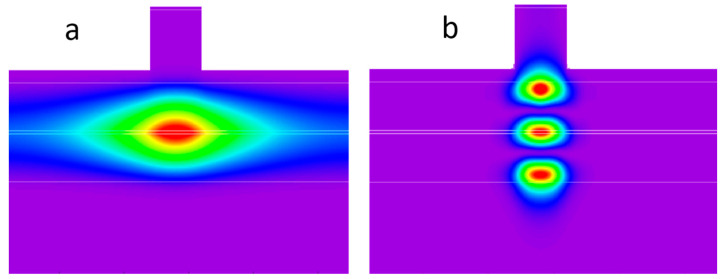
Calculated intensity contours for fundamental (**a**) and second-order (**b**) transverse modes in the Al_0.15_Ga_0.85_As/GaAs waveguide (ridge width 4 µm, waveguide thickness 1.7 µm, residual cladding thickness 50 nm). The active region locates in the center of the waveguide. Lateral and transverse sizes are in different scales.

**Figure 9:**
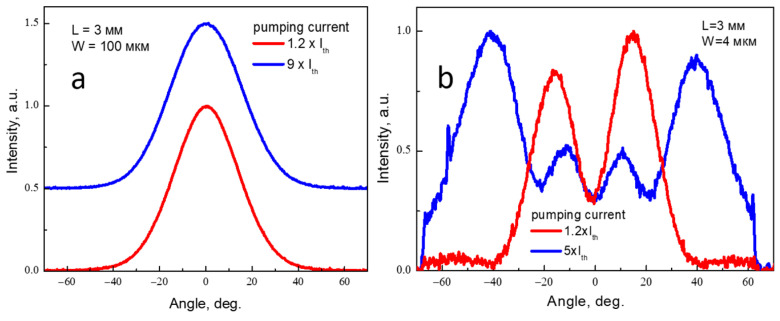
Transverse fundamental mode emission (**a**) of the broad-area laser and high-order mode emission (**b**) of the narrow-stripe laser processed from the same CLOC wafer.

Narrow-ridge lasers are usually designed to provide single-mode emission both in transverse and lateral directions. Typical lateral index-guided waveguides are formed by etching through the p-cladding. To reduce the optical loss and improve the laser reliability, the etching is stopped at a certain distance from the waveguide core. The residual cladding thickness provides an effective refractive index step required for the lateral mode guiding. The larger the optical mode penetration into the cladding, the higher the lateral effective refractive index step. The fundamental transverse mode is better localized within the core in comparison with the high-order modes, so the latter ones may have better lateral guiding (Figure 8), which promotes their lasing. This general consideration is relevant to the CLOC waveguides [27]. We have processed a CLOC wafer into broad-area lasers with 100 µm stripes and into 4 µm wide ridge lasers. The former ones showed stable emission on the fundamental transverse mode, while the latter evidently operated on the high-order modes (Figure 9). So, multimode or broadened transverse waveguides seem to be not so advantageous for use in spatial single-mode lasers. Designing such devices requires advanced 2D or even 3D simulations and should allow for a complete set of the optical modes, thermal and current-induced effects and the device imperfections caused by the epitaxial growth and the following processing. 

**Figure 10:**
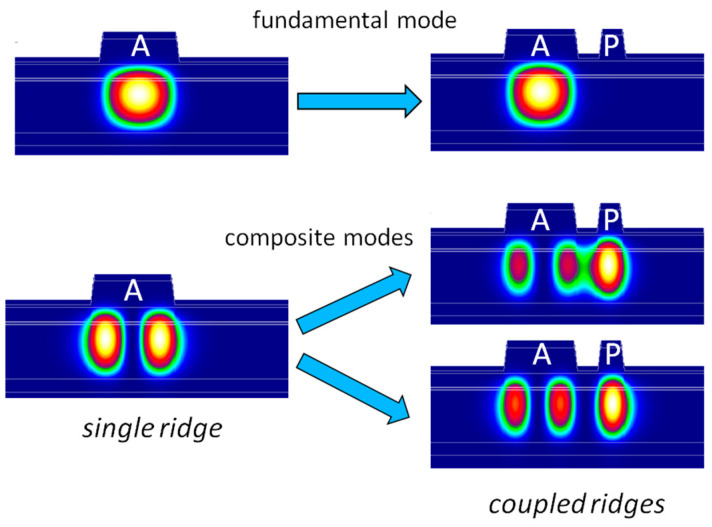
Simulated fundamental mode and composite modes in the two-stripe diode laser, where A—active ridge, P—passive ridge.

Up to now, we have discussed the CLOC concept being used for transverse waveguides where differences in the effective refractive indices for neighbor modes are defined by the waveguide contrast and can be as high as 0.05. This value is much higher than temperature- or current-induced refractive index variations. As a result, the CLOC ensures stable fundamental transverse mode operation. The same waveguide coupling formalism known as coupled mode theory [14] can be applied to transverse and lateral waveguides. Among the latter ones, directional couplers [14] and laterally coupled diode lasers are known [28]. As often as not for these devices, identical or nearly identical waveguides are employed. However, as we mentioned for the transverse CLOC basics, an effective coupling can be obtained for different waveguides if their modes have close effective refractive indices. In this sense, the CLOC concept has a potentiality for lateral mode engineering, for instance, for increasing ridge width in single-mode lasers by means of suppressing high-order lateral modes. Broader ridges would allow for obtaining higher output optical power. We have numerically treated the lateral CLOC design [29,30]. The lateral waveguide (Figure 10) consisted of a 10 µm active ridge and an optically coupled 4 µm passive single-mode ridge. The former guides two optical modes, but the first-order one resonantly tunnels into the passive waveguide and two composite modes are formed while the fundamental mode remains unchanged. As a result, this lateral CLOC waveguide system should support spatial single-mode lasing.

There were some attempts to implement similar ideas both numerically and experimentally. In [31], the authors have theoretically treated triple-ridge waveguide semiconductor lasers aimed at increasing the single-mode optical power. In these lasers, a broadened stripe waveguide is accompanied by a pair of lossy auxiliary waveguides, which in fact, reproduces the CLOC concept [30]. A theoretically obtained mode discrimination is as high as 10 cm^−1^. In work [31], the authors report the experimental results on suppressing lateral first-order mode in a stripe laser by neighboring single-mode passive waveguide. The obtained emission is quite far from the true single mode and worsens with increasing the pumping current. The major drawback of the lateral CLOC and similar designs [31,32] relates to the very low lateral waveguide contrast, which typically does not exceed 0.001. It means that the waveguide operation is very sensitive to the post-growth processing conditions and is highly affected by temperature- and current-induced refractive index changes. Thus, implementation of the lateral coupled-waveguide concept requires further extensive theoretical and experimental studies.

We believe that the effective use of the CLOC waveguide is not limited by broad-area lasers. For instance, transverse waveguides similar to those used in edge-emitting lasers are employed in microdisk lasers [33]. Due to the small diameter of 20–50 µm, the lasers operate at high injection current densities and have a rather large thermal resistance. This hard regime requires good heat dissipation, and any reduction of thermal resistance does matter. We have used the CLOC waveguide initially designed for edge-emitting lasers for microdisk ones [33]. The wafer comprises a 1.36-µm-thick undoped GaAs active waveguiding layer separated with a 0.25-µm-thick AlGaAs optical barrier from a 0.55-µm-thick GaAs passive waveguide. The p-side consists of a 0.5-µm-thick AlGaAs cladding and a 0.15-µm-thick GaAs contact layer. An active region containing five layers of InGaAs QDs is located within the waveguide core at a distance of 0.22 µm from the p-cladding. The total thickness of the semiconductor layers between the active region and the surface of the wafer is as low as 0.95 µm. The CLOC-based microlasers were bonded p-side down onto the Si boards and measured under the cw pumping current. To compare the key parameters, a set of reference microdisk lasers based on a standard transverse waveguide has been processed and measured. In the CLOC microlasers with a diameter of 31 µm, the thermal resistance was reduced to 0.32 K/mW in comparison with 0.59 K/mW measured for the standard microdisk lasers of the same diameter. Better heat dissipation allowed for increasing maximal optical powers from 0.83 mW to 1.92 mW. For the CLOC microdisk lasers tested, the peak power increased by 2.2–2.8 times.

**Figure 11:**
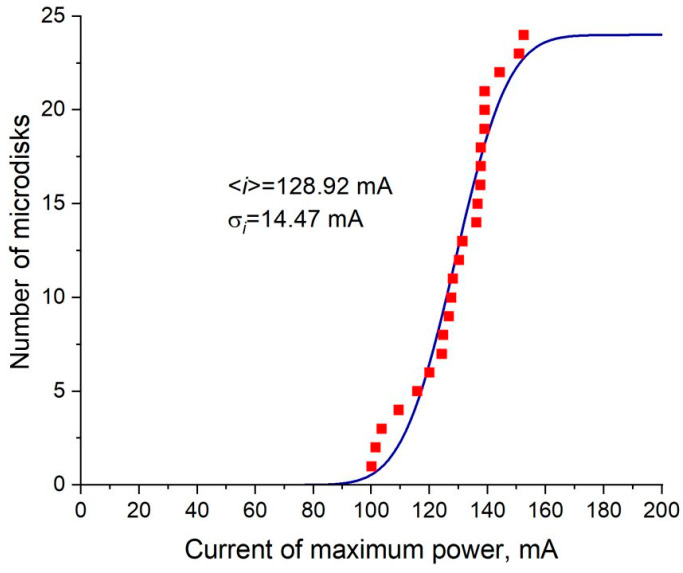
Number of microdisks among 24 devices with peak power current below the specified value: symbols-experiment, line-fit by a normal distribution.

Microdisk lasers based on a laser heterostructure with the CLOC waveguide demonstrate high uniformity of their characteristics and, in particular, high reproducibility of the injection current corresponding to the peak power. In microlasers, the maximum power is limited by the self-heating effect, and the current of the peak power depends on many device parameters that affect heat generation and its dissipation, such as threshold current, series resistance, turn-on voltage, thermal impedance, etc. For a group of 24 tested CLOC-microlasers with a diameter of 30 µm, we have found that the standard deviation of the peak current is only 11% of the mean value, As shown in Figure 11. 

## 6. Conclusions

To conclude, we have reviewed our CLOC concept proposed for optical mode engineering in laser planar waveguides. The approach is based on a well-known optical coupling phenomenon and allows for suppressing high-order parasitic modes and localizing the fundamental mode within the waveguide. The concept exploits the fact that only those optical modes of two waveguides interact intensively, the effective refractive indices of which are close to each other. The concept is often used in combination with shifting the active region toward the cladding layer. In view of this, a sufficient optical modal gain should be provided by the active region. The CLOC technology has been effectively employed for improving the characteristics of transverse waveguides in broad-area lasers. It enables the broadening of laser waveguides and significantly extends the range of allowed active region locations within the waveguide. Numerous results have shown that the CLOC approach leads to very stable fundamental mode lasing accompanied by reduced optical loss, as well as decreased thermal and electrical resistances (the latter is achieved owing to the possibility of decreasing the thickness of the *p*-type doped cladding layer). These parameters are crucial for high-power operations. Due to the strong localization of the fundamental transverse mode, the concept has limited applications for engineering lateral modes; however, this would be a subject for further research. Besides the edge-emitting lasers, we have successfully applied the CLOC waveguide for microdisk lasers, which has allowed reducing their thermal resistance and increasing the optical power by a factor of two at least. We believe that the CLOC approach is not limited to AlGaAs/GaAs-based lasers investigated and can be easily extended for other semiconductor compounds, which makes it rather simple and cost-efficient for the optical mode engineering and development of advanced high-power lasers.

## Data Availability

The data that support the findings of this study are available from the corresponding author upon reasonable request.
